# First Lines of the Practice of Physic

**Published:** 1785

**Authors:** 


### SECTION II.

### BOOKS.

I. *Firſt Lines of the Practice of Phyſic.* *By* William Cullen, *M. D. Profeſſor of the practice of Phyſic in the Univerſity of Edinburgh, Firſt Phyſician to His Majeſty for Scotland, Fellow of the Royal College of Phyſicians of Edinburgh, of the Royal Societies of London, of Edinburgh,* &c. &c. *Fourth edition, corrected*

*rected and enlarged*. 8vo. Elliot, *Edinburgh*, 1784. 4 vols.

IN his Preface to the prefent edition of this valuable work, the learned and experienced author offers fome remarks on the different fyftems of Phyfic that have hitherto been maintained, but more particularly on thofe of Stahl, Hoffman, and Boerhaave. The principal object of thefe remarks feems to be, to fhow that an humoral pathology has chiefly prevailed, and that this has conftantly given rife to an imperfect and erroneous theory.

In confidering the fyftem of Stahl, Dr. Cullen takes occafion to obferve, "that the "general doctrine of *Nature curing difeafes*, the "fo much vaunted Hippocratic method of "curing, has often had a very baneful influ-"ence on the practice of Phyfic; as either "leading Phyficians into, or continuing them "in, a weak and feeble practice; and, at the "fame time, fuperfeding or difcouraging all "the attempts of art."

In giving an account of Hoffman's fyftem, our author has chofen to deliver the fundamental principles of it in Hoffman's own words, which which he takes from the *Medicina Rationalis Syſtematica*. Tom. 3. ſect. 1. cap. 4.

In diſcuſſing the ſyſtem of Boerhaave, Dr. Cullen is at great pains to do juſtice to that illuſtrious profeſſor; but, at the ſame time, candidly points out the imperfections of what may be conſidered as fundamental doctrines of the Boerhaavian ſyſtem, and particularly the doctrine concerning the various ſtates of the fluids of the human body.

In the courſe of his Preface, Dr. Cullen enters into a critical examination of the late M. Lieutaud's *Synopſis Uuiverſæ Medicinæ*, and points out the numerous imperfections of that work.

The Preface cloſes with the following obſervations, which we chooſe to give in the author's own words, as they exhibit a ſummary view of the plan of the work. "I have en-"deavoured," ſays he, "to collect the facts "relative to the diſeaſes of the human body, as "fully as the nature of the work and the bounds "neceſſarily preſcribed to it would admit: but "I have not been ſatisfied with giving the "facts, without endeavouring to apply them to "the inveſtigation of proximate cauſes, and upon "theſe to eſtabliſh a more ſcientific and decided "method

" method of cure. In aiming at this, I flatter
" myfelf that I have avoided hypothefis, and
" what have been called *theories*. I have, in-
" deed, endeavoured to eftablifh many general
" doctrines, both phyfiological and patholo-
" gical; but I truft that thefe are only a gene-
" ralifation of facts, or conclufions from a cau-
" tious and full induction; and if any one fhall
" refufe to admit, or directly fhall oppofe, my
" general doctrines, he muft do it by fhowing
" that I have been deficient or miftaken in af-
" fuming and applying facts. I have, myfelf,
" been jealous of my being fometimes imper-
" fect in thefe refpects; but I have generally
" endeavoured to obviate the confequences of
" this, by proving, that the proximate caufes
" which I have affigned, are true in fact, as well
" as deductions from any reafoning that I may
" feem to have employed. Further, to obviate
" any dangerous fallacy in propofing a method
" of cure, I have always been anxious to fuggeft
" that which, to the beft of my judgement,
" appeared to be the method approved of by
" experience, as much as it was the confe-
" quence of fyftem.

" Upon this general plan I have endeavoured

" to form a fyftem of Phyfic that fhould com-

" prehend

"prehend the whole of the facts relating to the "science, and that will, I hope, collect and "arrange them in better order than has been "done before, as well as point out in particular "those which are still wanting to establish ge- "neral principles. This which I have at- "tempted, may, like other systems, hereafter "suffer a change; but I am confident that we "are at present in a better train of investigation "than Physicians were in before the time of Dr. "Hoffman. The affections of the motions "and moving powers of the animal œconomy, "must certainly be the leading inquiry in con- "sidering the diseases of the human body. "The inquiry may be difficult; but it must be "attempted, or the subject must be deserted al- "together. I have, therefore, assumed the "general principles of Hoffman, as laid "down in the passage which I have quoted "above: and if I have rendered them more "correct and more extensive in their applica- "tion; and, more particularly, if I have "avoided introducing the many hypothetical "doctrines of the humoral pathology, which "disfigured both his and all the other systems "which have hitherto prevailed; I hope I shall

"be

"be excuſed for attempting a ſyſtem, which "upon the whole may appear new."

In the body of the work, beſides many important additions, we meet with numerous corrections of ſtyle and arrangement, which render the whole more clear and explicit.—The proximate cauſe of fever, concerning which, the author, as is well known, has delivered a new and peculiar doctrine, is much more fully and ſatisfactorily ſtated in this than in the former editions. Dr. Cullen ſuppoſes, with Hoffman, that a ſpaſm of the extreme veſſels takes place in every fever; but he contends, that inſtead of being produced, as Hoffman imagines, by the remote cauſes of the fever, it is an effect of the *vis medicatrix naturæ*. The arguments for this he has placed in a new light.—We alſo meet with much additional matter concerning the doctrine of Atony in the extreme veſſels, and likewiſe concerning the difference of fevers and its cauſes.

In treating of the remote cauſes of fever, Dr. Cullen has added many new obſervations on the ſubject of cold, which he introduces by giving the curious and neceſſary diſtinction of the abſolute and relative powers of cold.

 The

The account of pneumonic inflammation is much improved in the prefent edition, and is followed by a new chapter on the peripneumonia notha, in which, after taking notice of the criticifm or rather cenfure paffed by M. Lieutaud on the hiftories of this difeafe as defcribed by Sydenham and Boerhaave, Dr. Cullen proceeds to defcribe it as he has obferved it, and as he fuppofes it to have appeared to Sydenham, Boerhaave, Van Swieten, Morgagni, and even to Lieutaud himfelf.

In the chapters on ophthalmia and hepatitis, among other additions, we have an account of the various remote caufes of thofe difeafes; and in treating of the latter difeafe, the author has added fome obfervations on the chronic hepatitis.

Thefe are fome of the principal improvements which we obferved on comparing attentively the firft volume of the new edition with the fame volume of the third edition. In the fecond and third volumes, the additions and corrections feem to be equally numerous and interefting.

The fourth volume begins with the fpafmodic affections of the natural functions, viz. *Pyrofis*, *Colic*, *Cholera*, *Diarrhœa*, *Diabetes*, *Hyfteria*,

*Hyfteria*, and *Hydrophobia*, which were included in the third volume of the former edition. In the remainder of the volume, which is new, the author, following the order obferved in his *Synopfis Nofologiæ Methodicæ*, treats firft of *Vefaniæ* or diforders of the intellectual functions. The particular difeafes comprehended under this clafs are diftinguifhed by Dr. Cullen according as they affect perfons in the time of waking or of fleeping. Thofe which affect men awake, he thinks may be confidered, as they confift in an erroneous judgement, to which he gives the name of *Delirium*; or, in a weaknefs or imperfection of judgment, which he names *Fatuity*. He begins with the confideration of delirium, and this, he obferves, may be fhortly defined — in a perfon awake, a falfe judgment arifing from perceptions of imagination, or from falfe recollection, and commonly producing difproportionate emotions. Such delirium, adds our author, is of two kinds; as it is combined with pyrexia and comatofe affections; or, as it is entirely without any fuch combination. It is the latter cafe that we term *Infanity*, and it is this kind of delirium that the author treats of in this part of his work.

We meet with a very ingenious and curious investigation of the cause of Infanity in general, in which the author makes great use of his doctrine concerning the different degrees of mobility and force of the nervous power. These different states, to which he applies the terms of *Excitement* and *Collapse*, are in no instance, he observes, more remarkable than in the different states of waking and sleeping. In the latter, when quite complete, the motion and mobility of the nervous power, with respect to the whole of what are called the animal functions, entirely cease, or, as Dr. Cullen expresses it, are in a state of collapse; and are very different from the state of waking, which, in healthy persons, he considers as a state of general and entire excitement.

As proofs that delirium may be, and frequently is, occasioned by an inequality in the excitement of the brain, Dr. Cullen instances the intermediate state of unequal excitement which takes place between sleeping and waking, when there almost always occurs more or less of delirium or dreaming. He farther observes, that the collapse in sleep is more or less complete, and that the most imperfect sleeps are those chiefly attended with dreaming; that dreams, dreams, therefore, moft commonly occur towards morning, when the complete ftate of fleep is paffing away; and farther, that dreams are generally excited by ftrong and uneafy impreffions made upon the body.—To this unequal excitement, our author is difpofed to attribute the phenomena of Infanity; and he thinks that the different ftates of denfity which have been obferved in the brains of maniacal fubjects by Morgagni, Meckel, and others, afford a confirmation of his general doctrine on this fubject.

Speaking of the ingenious attempts of Dr. Arnold, to diftinguifh the different fpecies of Infanity, as they appear with refpect to the mind, Dr. Cullen obferves, that the diftinctions pointed out by Dr. Arnold feem to be merely varieties, which can tend to little or no varieties of practice.—Our author contents himfelf with confidering the different ftates of infanity under the two heads of Mania and Melancholia; but he is fenfible that thefe two genera do not comprehend the whole of the fpecies of Infanity.

Dr. Cullen begins his account of mania or madnefs with a defcription of its fymptoms, and thefe he thinks, as well as the more ufual remote caufes of the difeafe, which he next confiders, point out a confiderable excefs and inequality in the excitement of the brain, and indicate the proper method of cure.

After fome remarks on the remedies commonly employed in maniacal cafes, Dr. Cullen proceeds to treat of melancholy, which has been commonly confidered as a partial infanity, and as fuch is defined in his nofology; but he now, it feems, entertains doubts whether this be altogether proper, and is difpofed to conclude, that the limits between general and partial infanity cannot always be fo exactly affigned, as to determine when the partial affection is to be confidered as giving a peculiar fpecies of difeafe, different from a more general infanity.

When infanity, neither ftrictly partial, nor entirely, nor conftantly general, occurs in perfons of a fanguine temperament, and is attended with agreeable rather than with angry or gloomy emotions, our author thinks fuch a difeafe muft be confidered as different from either the mania or melancholia. This laft, he obferves, muft be chiefly diftinguifhed by its occurring in perfons of melancholic temperament, and by its being always attended with fome feemingly groundlefs, but very anxious fear.

fear. As the ſame temperament, however, is common both to hypochondriaſis and melancholia, Dr. Cullen acknowledges he is at a loſs to determine how, in all caſes, the two diſeaſes may be diſtinguiſhed from one another.

The laſt claſs of diſeaſes which come under our author's conſideration is that of cachexies. The limits of our work will not allow us to follow him through the different diſeaſes arranged under this claſs; we ſhall, therefore, content ourſelves with quoting ſome of his opinions on ſcrophula and ſiphylis. The former of theſe he ſuppoſes to be commonly, and very generally, an hereditary diſeaſe, and to be derived more frequently from fathers than from mothers; but whether this happens from there being more ſcrophulous men than ſcrophulous women married, he is not certain. Dr. Cullen is of opinion, that this diſeaſe depends on a peculiar conſtitution of the lymphatic ſyſtem, but he oppoſes the doctrine of thoſe writers who have ſuppoſed it to be derived from the venereal diſeaſe.

Dr. Cullen obſerves, that for the cure of ſcrophula, we have not yet learned any mode of treatment that is certainly, or even generally ſucceſsful. As mineral waters of almoſt every every kind have been occaſionally of uſe in this diſeaſe, he ſuſpects that the elementary water is the chief part of the remedy. In ſea water he has diſcovered no ſuperior efficacy.— He has ſeen no immediate cure produced by the Peruvian bark; but in ſeveral inſtances he has experienced the good effects of the expreſſed juice of coltsfoot. Hemlock he has ſometimes found of uſe in diſperſing obſtinate ſwellings; but in this alſo it has often diſappointed him; and he has not, at any time, obſerved that it diſpoſed ſcrophulous ulcers to heal. He has never found either mercury or antimony, in any ſhape, of uſe in this diſeaſe; and when any degree of a feveriſh ſtate has come on, the uſe of mercury, he obſerves, has proved manifeſtly hurtful. Cold bathing, it ſeems, has been of more benefit than any other remedy he has had occaſion to ſee employed.

When ſcrophulous tumours are advancing towards ſuppuration, Dr. Cullen cautions us againſt haſtening it by the application of poultices, becauſe theſe ſwellings ſometimes diſappear ſpontaneouſly, but never after they have begun to inflame, and he is of opinion, that the ſcrophulous matter is rendered more acrid by communication with the air.

In

In the treatment of fcrophulous ulcers, Dr. Cullen obferves, that efcharotic preparations have feldom fucceeded, but more commonly have caufed the ulcer to fpread more. He recommends linen cloths wetted with cold water in the day time, and a cloth fpread with a mild ointment at night, as the beft applications to fores of this fort. He has generally found fea water too irritating.

On the fubject of fyphilis, our author profeffes to confine himfelf to fuch general remarks as may tend to illuftrate fome parts of the pathology, or of the practice. He is convinced, that the infection producing Gonorrhœa, and that which produces chancres and fyphilis, are one and the fame. He fuppofes Gonorrhœa to be, in general, a local difeafe, depending on an inflamed ftate of the urethra, which may often exift without communicating any infection to the other parts of the body; but when the inflammation extends to a certain degree, fuppuration and ulcer are produced, and a general fyphilis may take place.

Dr. Cullen has met with inftances of difcharges from the urethra, refembling thofe of a virulent Gonorrhœa, in perfons in whom there was no reafon to fufpect any venereal infection; and ſuch a virulent appearance, we are told, frequently occurs in perſons labouring under a gleet, without any freſh infection having taken place ; intemperance in venery and drinking, are mentioned as the moſt frequent cauſes of this appearance.

In moſt caſes of Gonorrhœa, Dr. Cullen ſuppoſes the practice of applying opium to the urethra, and of exhibiting it by the mouth, to be extremely uſeful. — When the inflammatory ſymptoms are much abated, he recommends injections of moderate aſtringency and likewiſe mercurial injections. Of the latter, that which he has found moſt uſeful is a ſolution of corroſive ſublimate in water ; ſo much diluted as not to occaſion any violent ſmarting, but not ſo much diluted as to excite no ſmarting at all. From mucilaginous or oily injections during the inflamed ſtate of the urethra, he has ſeldom experienced much benefit.

In every caſe of chancre, Dr. Cullen ſuppoſes the internal uſe of mercury to be neceſſary. He is an advocate for the early healing of chancres, as the ſymptoms of general ſyphilis have generally ſeemed to him to be more conſiderable in proportion to the length of time ulcers of this kind have been ſuffered to remain unhealed.

unhealed. He mentions the application of red precipitate in a dry powder as the moſt powerful means of healing them.

With regard to the various preparations of mercury, our author ſuppoſes the choice of them to be, for the moſt part, a matter of indifference. The proper adminiſtration of mercury, he is of opinion, conſiſts, firſt, in the chooſing ſuch preparations of it as are the leaſt diſpoſed to run off by ſtool, for which reaſon, in general, he ſeems to prefer mercurial frictions; ſecondly, in employing mercury till the mouth is ſenſibly affected; and thirdly, in continuing the uſe of it for ſome time after all the ſymptoms of the diſeaſe have diſappeared.

Dr. Cullen obſerves, that corroſive ſublimate has often been employed with advantage; but he thinks it requires to be continued for a longer time than the other preparations of mercury; and he ſuſpects, alſo, that it has often failed of producing a cure, owing to the patient's having been expoſed to the free air during its uſe.

Dr. Cullen is perſuaded, that in moſt caſes, mercury, properly employed, will prove a very certain and effectual remedy; and with reſpect to other remedies that have been propofed, he contents himfelf with obferving, that he has found the decoction of the Mezereon contribute to the healing of ulcers, which feemed to have refifted the power of mercury.

