# Errata

**Published:** 1785

**Authors:** 


					-p. 6z, 1 13, ior
tower/#, read umyerj*.?

				

